# Publisher’s Note: Continued Publication of *Pathophysiology*, the Official Journal of the International Society of Pathophysiology, by MDPI

**DOI:** 10.3390/pathophysiology27010001

**Published:** 2020-09-22

**Authors:** Franck Vazquez

**Affiliations:** MDPI, St. Alban-Anlage 66, CH-4052 Basel, Switzerland; vazquez@mdpi.com

*Pathophysiology* (ISSN 0928-4680) was launched in 1994 and has been published during the past 26 years by Elsevier [1,2] as the official journal of the International Society of Pathophysiology (ISP). The ISP was founded in 1991 with a mission to create a platform for global networking in the area of pathophysiology and to foster the versatile development of this branch of physiology [3]. Initially, *Pathophysiology* was intended to be discontinued in December 2019 [4], but thanks to the kind and generous support of Elsevier, we are very happy to be able to continue the publication of *Pathophysiology* under the same ISSN and ensure that the legacy of the ISP continues.

We are extremely honored to work closely with Professor J. Steven Alexander [5], who has been the Editor-in-Chief of *Pathophysiology* for more than five years, with Professor Olga Pechanova, the President of the ISP [6], and with the members of the editorial board to continue to publish significant and impactful articles in this research field.

MDPI’s portfolio has greatly expanded in the last few years, and today sums up to 250 journals, in all fields and disciplines [7], including more than 50 journals relevant to the biomedical domain. We are delighted to add *Pathophysiology* to our biomedical journal portfolio and continue to serve this scientific community well.

We will publish one quarterly issue in 2020 and regularly publish four quarterly issues as of 2021.

Enjoy publishing in *Pathophysiology*!
